# Association of the G473A Polymorphism and Expression of Lysyl Oxidase with Breast Cancer Risk and Survival in European Women: A Hospital-Based Case-Control Study

**DOI:** 10.1371/journal.pone.0105579

**Published:** 2014-08-20

**Authors:** Andrea Friesenhengst, Tamara Pribitzer-Winner, Martin Schreiber

**Affiliations:** 1 Department of Obstetrics and Gynecology, Medical University of Vienna, Vienna, Austria; 2 University of Applied Sciences, Vienna, Austria; 3 Comprehensive Cancer Center, Medical University of Vienna, Vienna, Austria; Taipei Medical University, Taiwan

## Abstract

**Background:**

Lysyl oxidase (LOX) is an extracellular enzyme essential for the covalent crosslinking of extracellular matrix proteins and may also have additional functions. LOX expression can be both up- and downregulated in cancer and is associated both with tumour suppression and metastasis progression. The G473A polymorphism (rs1800449) results in the Arg158Gln amino acid substitution in the LOX propeptide, compromises its tumour suppressive activity, and was associated with an increased breast cancer risk in a Chinese Han population. In the first hospital-based case-control study in European women, we aimed at investigating the association of LOX expression and the G473A polymorphism with breast cancer risk and survival in unselected and estrogen receptor (ER) negative patients.

**Methodology/Principal Findings:**

The G473A polymorphism was genotyped in 386 breast cancer patients and 243 female controls. Moreover, LOX mRNA expression was quantified in the tumors of 105 patients by qRT-PCR. We found that the minor A-allele of this polymorphism is associated with a later age at breast cancer onset, a trend towards a decreased disease-free and metastasis-free survival, but not with an increased breast cancer risk. LOX mRNA expression was significantly elevated in tumours of patients older than 55 years, postmenopausal patients, estrogen receptor positive tumours, and p53 negative tumours, but was unaffected by G473A genotype in tumours and breast cancer cell lines. High LOX expression was associated with a poor disease-free and metastasis-free survival in ER negative but not ER positive patients. LOX expression was an independent prognostic parameter in multivariate analysis, whereas G473A genotype was not. A small, distinct subgroup of the ER negative patients was identified which exhibited a considerably elevated LOX expression and a very poor disease-free (p = 0.001) and metastasis-free survival (p = 0.0003).

**Conclusions/Significance:**

This newly identified ER negative/LOX high subgroup may be a suitable collective for future individualized breast cancer diagnosis and therapy.

## Introduction

Lysyl oxidase (LOX) is a secreted copper-dependent amine oxidase, which catalyses the oxidative deamination of lysine and hydroxylysine residues to aldehydes, thus initiating the covalent crosslinking of collagens and elastin in the extracellular matrix (ECM) [1], [2]. LOX may also have other functions in addition to ECM maturation, and may enzymatically modify additional non-ECM proteins [1]. The *LOX* gene is located at chromosome 5q23.2 and codes for a 50 kDa inactive proenzyme (Pro-LOX), which is secreted and then proteolytically cleaved by bone morphogenic protein 1 (BMP1) into a 32 kDa active enzyme (LOX) and an 18 kDa propeptide (LOX-PP) [1]. *LOX* is a member of a family of lysyl oxidases which also includes four additional paralogues, *LOXL1–4* [1]. Aberrant expression of these enzymes is associated with a number of human diseases, especially cancer. LOX expression is induced by hypoxia-inducible factor (HIF) through a hypoxia-responsive element in the *LOX* promotor, and is associated with hypoxia in breast tumours [3]. Patients with highly hypoxic tumours tend to have a poor overall and metastasis-free survival [3]. Interestingly, LOX has a dual role in cancer both as a tumour suppressor and as a metastasis promoter [1], [4]. The precise function of the LOX family members in tumorigenesis appears to depend on cellular location, cell type and transformation status of the tumour in which they are expressed. Reduced LOX expression has been observed in many carcinomas, and the ectopic expression of LOX inhibited tumour progression in several experimental model systems [4]–[7]. For example, LOX inhibited the transforming activity of HRAS in NIH 3T3 fibroblasts and its initial name hence was “ras recision gene (*rrg*)” [1], [4], [8].

On the other hand, increased expression of LOX and LOXL2 is associated with aggressive tumours, decreased survival, and increased metastasis in cancer of the colon, breast, lung, prostate, and others [1], [3]. High LOX expression was associated with a significantly shorter metastasis-free and overall survival in breast cancer patients with estrogen receptor (ER) negative, but not ER positive tumours, as well as in head and neck cancer patients [3]. The effects of LOX on growth, invasiveness and migration appear to be more important in metastatic growth than in primary tumour formation, exemplified by pronounced effects of LOX inhibition on metastasis formation, but not on primary tumour growth in an orthotopic xenotransplantation model [3]. Mechanistically, LOX family members have been suggested to promote metastasis by modulating the extracellular matrix surrounding the tumour, which can lead to the activation of focal adhesion kinase (FAK), SRC, MAPK, and integrins, as well as by the induction of an epithelial-mesenchymal transition (EMT) via SNAI1 [1]. Moreover, secreted LOX is involved in the recruitment of inflammatory cells to distant sites, which contributes to metastasis by initiating the formation of the premetastatic niche [9].

LOX has both intracellular and extracellular functions, and the tumour suppressive activity appears to be predominantly due to its intracellular functions, particularly the modulation of gene expression [1]. In contrast, extracellular LOX is mostly pro-tumorigenic and pro-metastatic, particularly via remodelling the extracellular matrix of the tumour microenvironment [1]. Moreover, the tumour suppressor activity of LOX is executed, at least in part, by the pro-peptide, which inhibited tumour formation and the invasiveness of HER2/neu driven NF639 breast cancer cells [4], [10], [11]. The LOX-PP is also capable of inhibiting NF-κB, a key pro-tumorigenic transcription factor [1], [12].

The G473A (rs1800449) single nucleotide polymorphism (SNP) is located in a highly conserved region of this propeptide domain of *LOX*, and results in the amino acid substitution Arg158Gln [4]. Presence of the Gln-variant, which is encoded by the minor A-allele, was found to profoundly impair the tumour suppressive activity of the LOX-PP in a xenograft model of NF639 breast cancer cells [4]. The A-allele of this SNP is associated with an increased risk of osteosarcomas and ovarian, gastric, and non-small cell lung cancer [13]–[17]. Moreover, the AA-genotype was also associated with a significantly shorter survival in non-small cell lung cancer [14]. Association of the G473A SNP with breast cancer risk has been analysed in two case-control studies thus far [4], [18]. The A-allele was associated with a considerably increased risk of breast cancer in a study of a Chinese Han population involving 238 breast cancer patients and 234 controls [18]. In contrast, no increased risk was observed in a study of African American women (311 patients and 446 controls), although a possible trend towards an increased risk of ER negative breast cancer associated with the A-allele was suggested by these authors [4].

Metastasis is the major cause of cancer morbidity and mortality. Accordingly, it is important to characterize metastasis promoters such as LOX in the context of survival and established clinical and histopathological characteristics of human cancer. Moreover, the G473A loss-of-function polymorphism is an obvious candidate to affect breast cancer risk, but has only been analysed in two non-European study populations thus far [4], [18]. Accordingly, the overall aim of our work was a detailed analysis of the association of LOX mRNA expression and the G473A SNP with clinically relevant properties of human breast cancer such as prognosis, age at onset, and clinical and histopathological characteristics of routine breast cancer categorization and staging such as the ER status. Moreover, we wanted to analyse the G473A SNP in a European breast cancer study population for the first time.

## Results

### The LOX G473A SNP and Breast Cancer Risk

A coding SNP in the *LOX* gene (rs1800449; c.473G>A; Arg158Gln; R158Q; hereafter referred to as G473A) was genotyped in a hospital-based case-control study of 386 breast cancer patients and 243 female controls. Clinical characteristics of the study population, together with the frequency of the G473A genotypes in the study population and its subpopulations are shown in Table S1. Both the control population (*p* = 0.76) and the patient population (*p* = 0.42) were in Hardy-Weinberg equilibrium. The frequency of the minor A-allele was 14.9% in patients and 15.6% in controls. The frequencies of the genotypes GG, GA and AA were 73.1%, 24.1% and 2.8% in patients, and 71.6%, 25.5% and 2.9% in controls, respectively (Table S1). These genotype and allele frequencies are very similar to those reported in the HapMap-CEU database of healthy individuals of European ancestry (GG, 69.0%; GA, 28.3%; AA, 2.7%; n = 226).

Dominant, recessive and log-additive inheritance models for the association of *LOX* G473A genotypes with breast cancer risk in the study population and in clinically relevant subgroups were analysed (Table 1). No significant associations of the G473A SNP with breast cancer risk were observed in our study population or subpopulations thereof in any of the three inheritance models. Additional comparisons of G473A genotypes and A *vs.* G alleles were also analysed, and odds ratios and 95% confidence intervals adjusted for age and menopausal status as well as unadjusted values were determined (Table S2). All these comparisons revealed odds ratios close to unity, and none of the investigated genotypes or alleles was significantly associated with an increased breast cancer risk. Unlike a previous study of African American women [4], we did not observe a trend towards an association of the minor allele with increased risk of estrogen receptor (ER)-negative breast cancer in our European study population (Table 1).

**Table 1:** Association of the *LOX* G473A polymorphism with breast cancer risk in the indicated inheritance models and subgroups.

			dominant		recessive		log-additive	
	Subgroup	No. of cases (%)	OR (95% CI)	p-value	OR (95% CI)	p-value	OR (95% CI)	p-value
**All patients**		386 (100%)	0.93 (0.65–1.33)	0.692	0.99 (0.38–2.59)	0.982	0.95 (0.70–1.29)	0.727
**Age (years)** a	<55	164 (42.5%)	0.73 (0.46–1.16)	0.186	0.84 (0.24–2.93)	0.786	0.78 (0.52–1.16)	0.219
	≥55	222 (57.5%)	1.09 (0.73–1.63)	0.673	1.10 (0.38–3.18)	0.864	1.08 (0.76–1.52)	0.675
**Menopausal status**	pre	95 (28.5%)	0.76 (0.44–1.32)	0.324	0.36 (0.04–2.96)	0.283	0.75 (0.45–1.23)	0.239
	post	238 (71.5%)	1.07 (0.72–1.59)	0.729	1.17 (0.42–3.29)	0.762	1.07 (0.76–1.50)	0.691
**Tumour type**	ductal	233 (74.9%)	0.89 (0.63–1.27)	0.531	0.89 (0.29–2.69)	0.838	0.89 (0.63–1.27)	0.531
	lobular	78 (25.1%)	1.34 (0.78–2.30)	0.301	0.89 (0.18–4.36)	0.882	1.23 (0.77–1.97)	0.393
**Tumour size**	pT1	168 (53.0%)	0.92 (0.59–1.43)	0.720	1.03 (0.32–3.32)	0.955	0.95 (0.65–1.38)	0.772
	pT2–4	149 (47.0%)	1.09 (0.70–1.71)	0.703	1.17 (0.36–3.76)	0.792	1.08 (0.74–1.59)	0.679
**Stage**	0 or I	135 (43.1%)	0.75 (0.46–1.23)	0.248	0.77 (0.19–3.01)	0.699	0.78 (0.51–1.20)	0.260
	II-IV	178 (56.9%)	1.07 (0.70–1.64)	0.758	1.18 (0.39–3.56)	0.775	1.07 (0.74–1.54)	0.720
**Grade**	pG1–2	226 (62.1%)	0.89 (0.59–1.34)	0.578	0.92 (0.30–2.78)	0.882	0.91 (0.64–1.29)	0.597
	pG3	138 (37.9%)	0.89 (0.56–1.43)	0.627	1.27 (0.39–4.07)	0.693	0.94 (0.63–1.41)	0.779
**Lymph node status**	pN0	184 (59.4%)	0.84 (0.54–1.30)	0.433	0.75 (0.22–2.60)	0.645	0.85 (0.58–1.25)	0.408
	pN+	126 (40.6%)	0.97 (0.60–1.57)	0.900	1.11 (0.32–3.85)	0.875	0.99 (0.65–1.49)	0.955
**ER status**	pos	232 (67.6%)	1.02 (0.69–1.52)	0.907	0.90 (0.30–2.70)	0.844	1.01 (0.71–1.42)	0.968
	neg	111 (32.4%)	0.85 (0.51–1.42)	0.533	1.26 (0.36–4.40)	0.720	0.91 (0.59–1.41)	0.682
**PR status**	pos	160 (47.5%)	0.99 (0.63–1.54)	0.953	1.09 (0.34–3.49)	0.888	1.00 (0.68–1.46)	0.996
	neg	177 (52.5%)	0.94 (0.61–1.45)	0.773	0.98 (0.31–3.14)	0.973	0.95 (0.65–1.38)	0.795
**HER2 status**	pos	68 (20.0%)	1.21 (0.68–2.15)	0.529	1.02 (0.21–5.03)	0.979	1.15 (0.70–1.90)	0.581
	neg	272 (80.0%)	0.90 (0.61–1.32)	0.577	1.03 (0.37–2.87)	0.962	0.92 (0.66–1.29)	0.643
**p53 status**	pos	88 (26.1%)	0.65 (0.36–1.17)	0.140	0.78 (0.16–3.85)	0.760	0.70 (0.42–1.18)	0.168
	neg	249 (73.9%)	1.09 (0.74–1.60)	0.674	1.12 (0.40–3.14)	0.830	1.08 (0.77–1.50)	0.666

Dominant model, GA+AA vs. GG; recessive model, AA vs. GG+GA; log-additive model, GG = 0, GA = 1, AA = 2; OR, odds ratio; 95% CI, 95% confidence interval; ER, estrogen receptor; PR, progesterone receptor; pN0, no lymph node metastases; pN+, patients with lymph node metastases.

a patients aged under 55 years or ≥55 years at diagnosis were compared to control subjects of any age for sake of comparability with the other subgroup analyses.

### Association of the LOX G473A SNP with Age at Breast Cancer Onset

**Figure 1:**
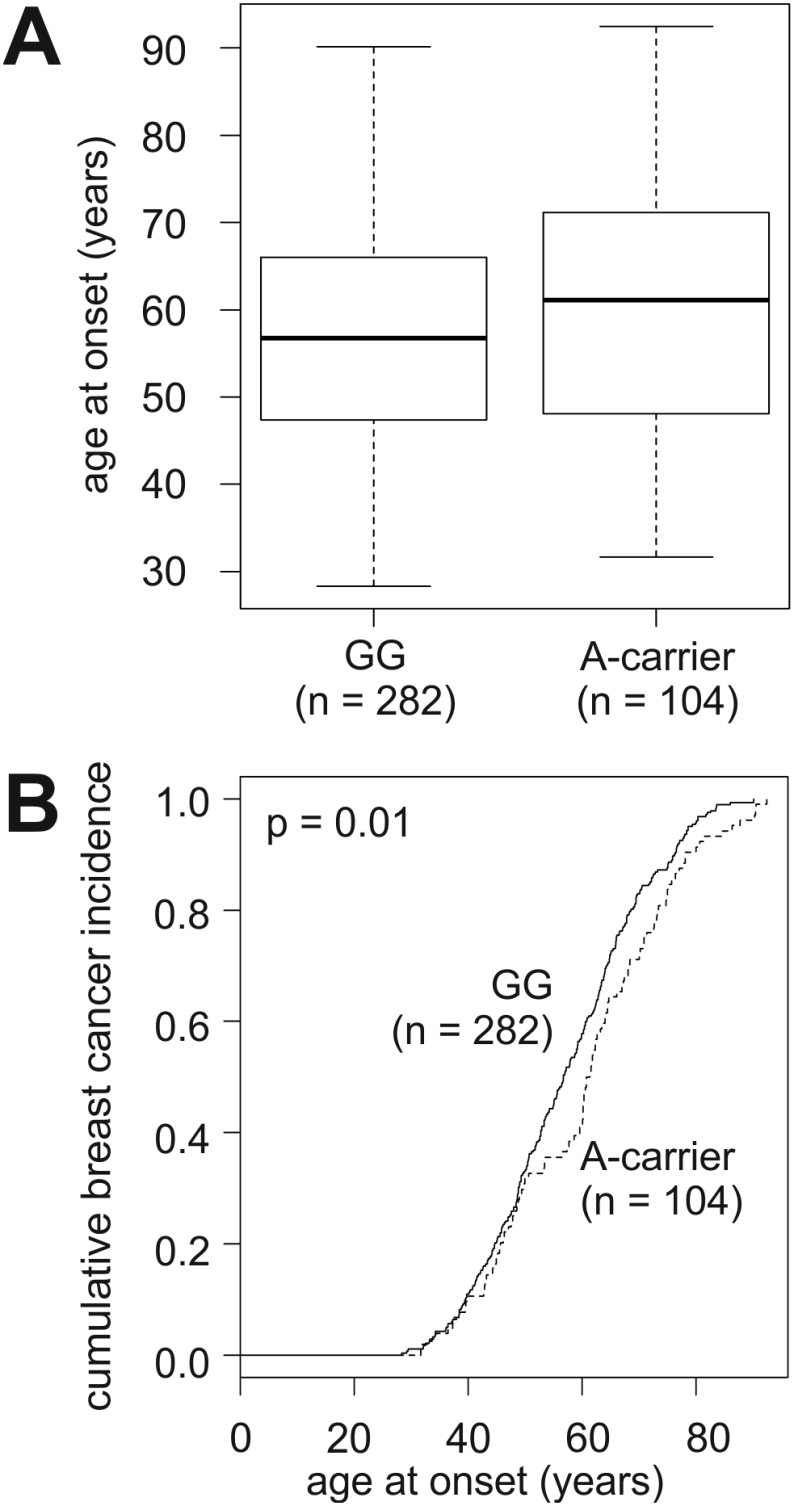
Age at breast cancer onset as a function of *LOX* G473A genotype. (A) Boxplot of the age at onset of patients with the homozygous genotype GG and of A-carriers. p = 0.04, unpaired two-sided t-test. (B) Curves of the cumulative breast cancer incidence at the indicated age at onset of GG patients and of A-carriers. p = 0.01, log-rank test. A-carrier, patients with the GA (n = 93) or AA (n = 11) genotype.

Next, we investigated the potential impact of the G473A SNP on the age at breast cancer onset. Since the number of patients with the AA genotype was small (n = 11; Table S1), they were pooled with the GA patients for these analyses, and the resulting group of A-carriers (n = 104) were compared to the major genotype GG (n = 282). A-carriers were diagnosed with breast cancer at a mean age of 60.5±14.8 years (median, 61.1), and GG patients at 57.1±13.3 years (median, 56.8; Fig. 1A). Thus, A-carriers had a significantly later mean age at onset (p = 0.04, t-test). GG patients and A-carriers had roughly the same cumulative breast cancer incidence up to an age of 50, however, the curve of A-carriers considerably lagged behind thereafter (Fig. 1B). This “lag-phase” of A-carriers occurred at an age of 50–60, which roughly coincides with menopause. As a consequence, A-carriers attained any cumulative incidence rate approximately 4–6 years later than GG patients after that age, a difference that remained rather constant until an age >85 (Fig. 1B). Thus, the overall cumulative incidence of A-carriers differed significantly from that of GG patients (p = 0.01, log-rank test). Comparison of all three G473A genotypes also revealed significant differences in their cumulative breast cancer incidence (p = 0.007, log-rank test; data not shown).

### Association of the LOX G473A SNP with Breast Cancer Prognosis

**Figure 2:**
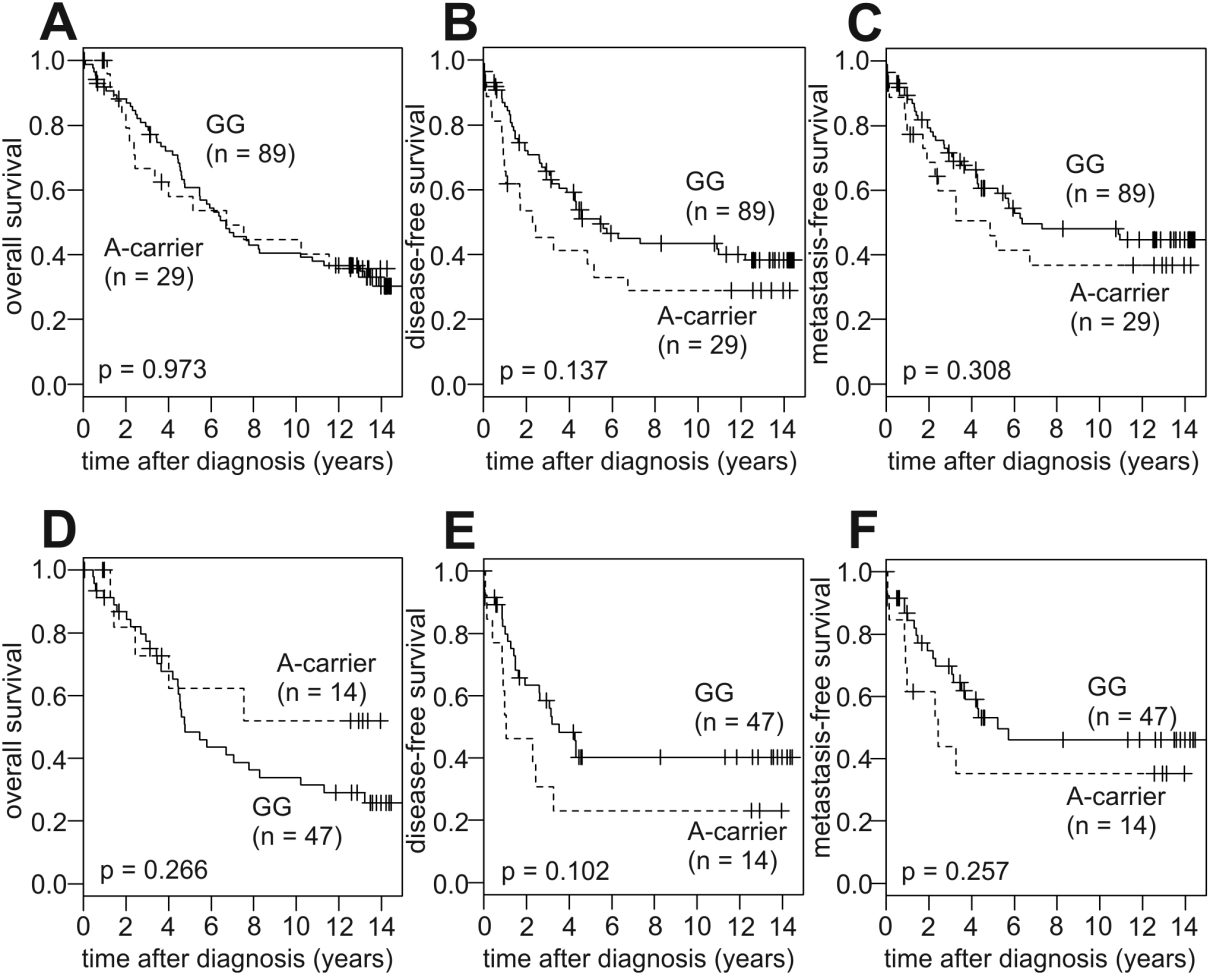
Association of *LOX* G473A genotypes with the survival of breast cancer patients. Kaplan-Meier analyses of the overall (A, D), disease-free (B, E) and metastasis-free survival (C, F) in unselected patients (A–C; n = 118) and in ER-negative patients (D–F; n = 61) are shown. A-carrier, patients with the AG (n = 26) or AA genotype (n = 3; 2 ER pos and 1 ER neg).

Detailed follow-up records were available for 118 of the genotyped patients. We subjected these patients to Kaplan-Meier analyses of the overall (OS), disease-free (DFS), and metastasis-free survival (MFS), comparing A-allele carriers with patients with the homozygous GG genotype (Fig. 2). We grouped AA patients (n = 3) together with GA patients (n = 26) since their number was too small for a separate analysis. These Kaplan-Meier analyses were performed in the entire population (Fig. 2A–C), and separately in ER negative patients (Fig. 2D–F) and in ER positive patients (Fig. S1G–I). No significant differences in the survival of A-carriers vs. GG patients were observed. However, there was a clear trend towards a decreased disease-free survival of A-carriers in unselected (p = 0.14) and, more pronounced, in ER negative patients (p = 0.1; Fig. 2B, E). A similar, but weaker trend was found for the metastasis-free survival (Fig. 2C, F). In contrast, no such trend was observed for the OS, or for the DFS or MFS in ER positive patients (Fig. 2A, D and Fig. S1G–I).

### Analysis of LOX G473A Genotype and LOX Expression in Breast Cancer and Control Cell Lines

**Figure 3:**
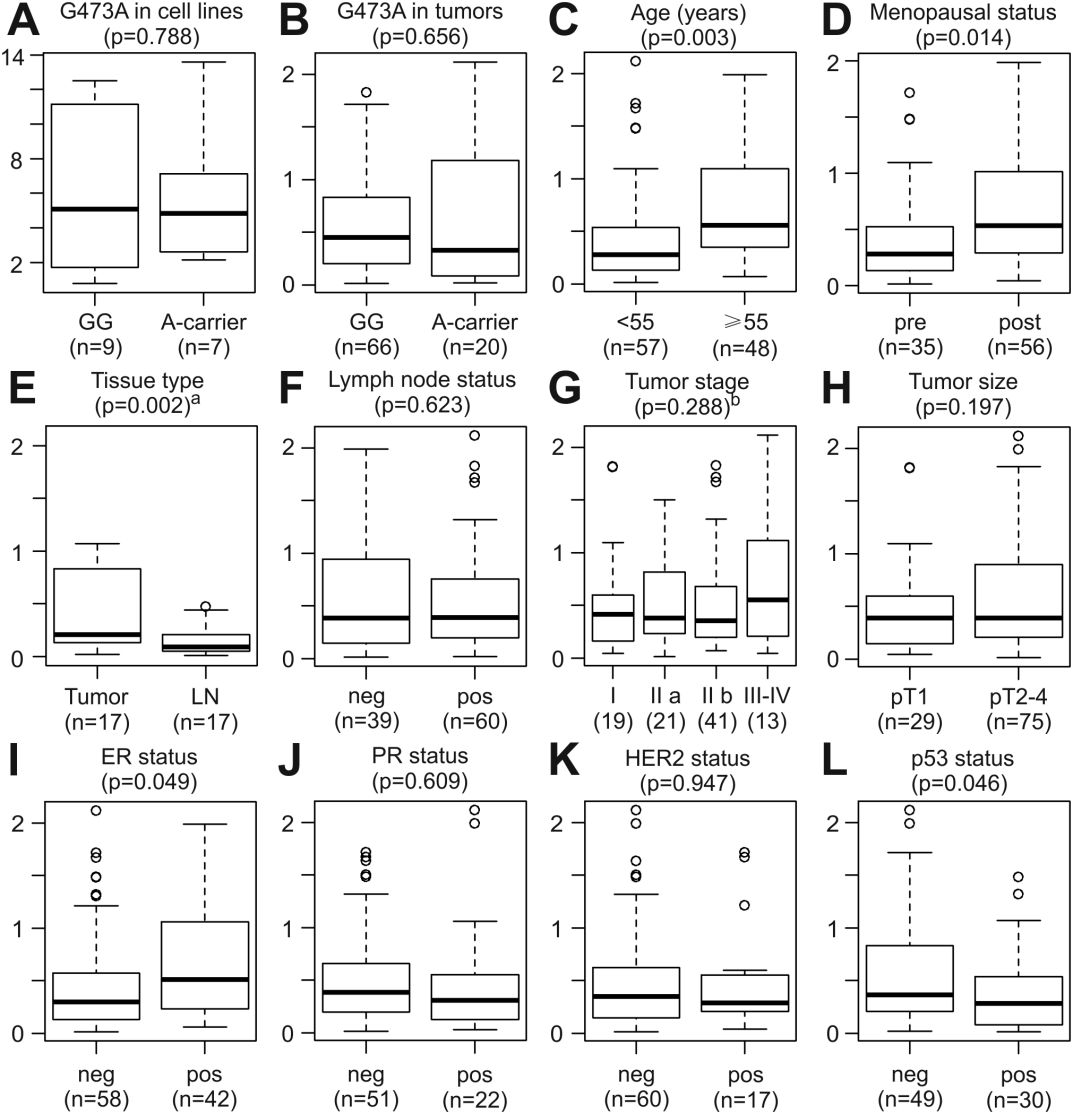
LOX expression in breast tumors and breast cancer cell lines. Correlation of relative LOX expression (2^−ΔΔCt^ values; y-axes) with *LOX* G473A genotype and with established clinical and histopathological parameters was analysed in breast cancer cell lines (A) and in breast tumors (B–L). All p-values were determined via unpaired, two-sided t-tests except in E^a^. (A) Boxplot of LOX expression in breast cancer cell lines with the indicated G473A genotypes. Note the different range of the y-axis compared to B-L, and that log(2) expression values (−ΔΔCt values) are shown in A. (B) LOX expression in breast tumours from patients with the indicated G473A genotypes. A-carrier, patients with the AG (n = 17) and AA (n = 3) genotype. (C) LOX expression in patients with an age at breast cancer onset of <55 vs. ≥55 years. (D) LOX expression in pre- vs. post-menopausal patients. (E) LOX expression in paired tissue specimens of primary tumours vs. lymph node metastases (LN) of 17 patients. Accordingly, the indicated p-value of 0.002 was determined by paired, two-sided t-test. (F) LOX expression in breast tumours from patients with a negative (neg, pN0) vs. a positive (pos, pN+) lymph node status. (G) LOX expression in patients with the indicated tumor stages. Numbers in parentheses indicate the number of patients in each group. (H) LOX expression in pT1 vs. pT2–4 breast tumours. (I) LOX expression in estrogen receptor (ER) neg vs. pos tumours. (J) LOX expression in progesterone receptor (PR) neg vs. pos tumours. (K) LOX expression in HER2 neg vs. pos tumours. (L) LOX expression in p53 neg vs. pos tumours. ^a^p-value was determined by paired, two-sided t-test in E; ^b^p-value of an unpaired, two-sided t-test of stage I–II vs. stage III–IV patients is shown in G. LN, lymph node metastases; neg, negative; pos, positive.

We also analysed the G473A SNP in 16 human breast cancer cell lines, and detected the genotype GG in nine cell lines (Cama1, Hcc1143, Hcc1937, Kpl1, MCF7, MDA-MB231, MDA-MB435, MDA-MB453, MDA-MB468), GA in four cell lines (AU565, BT474, SKBR3, T47D), and AA in three cell lines (Cal51, Hs578T, ZR75-1). All three untransformed breast epithelial cell lines that were genotyped in parallel (HMEC, MCF10A, MCF10F) exhibited the genotype GG. Genotyping of seven of these cell lines (MDA-MB231, MCF7, MCF10A, BT474, SKBR3, T47D, and Hs578T) has been reported previously, with 100% concordance to our genotype results [4]. In summary, 25% (4/16) of these breast cancer cell lines exhibited the genotype GA, and 18.8% (3/16) the genotype AA. Thus, the frequency of the minor A-allele was 31.3% in these cell lines, substantially above the 15.2% observed in our study population (Table S1), as well as the 16.7% global minor allele frequency reported in the HapMap database, as pointed out previously [4]. We next determined the relative expression of LOX in these cell lines via qRT-PCR, and correlated the results with the G473A genotypes (Fig. 3A). These 16 breast cancer cell lines exhibited a rather large variation in LOX expression levels, but no significant differences were observed between cell lines with the GG genotype vs. A-carriers (p = 0.79, t-test; Fig. 3A). A significant association of G473A genotypes with LOX expression was not observed in tumour samples either (Fig. 3B; see below).

### Association of LOX Expression with Clinical and Histopathological Characteristics of Breast Cancer

**Figure 4:**
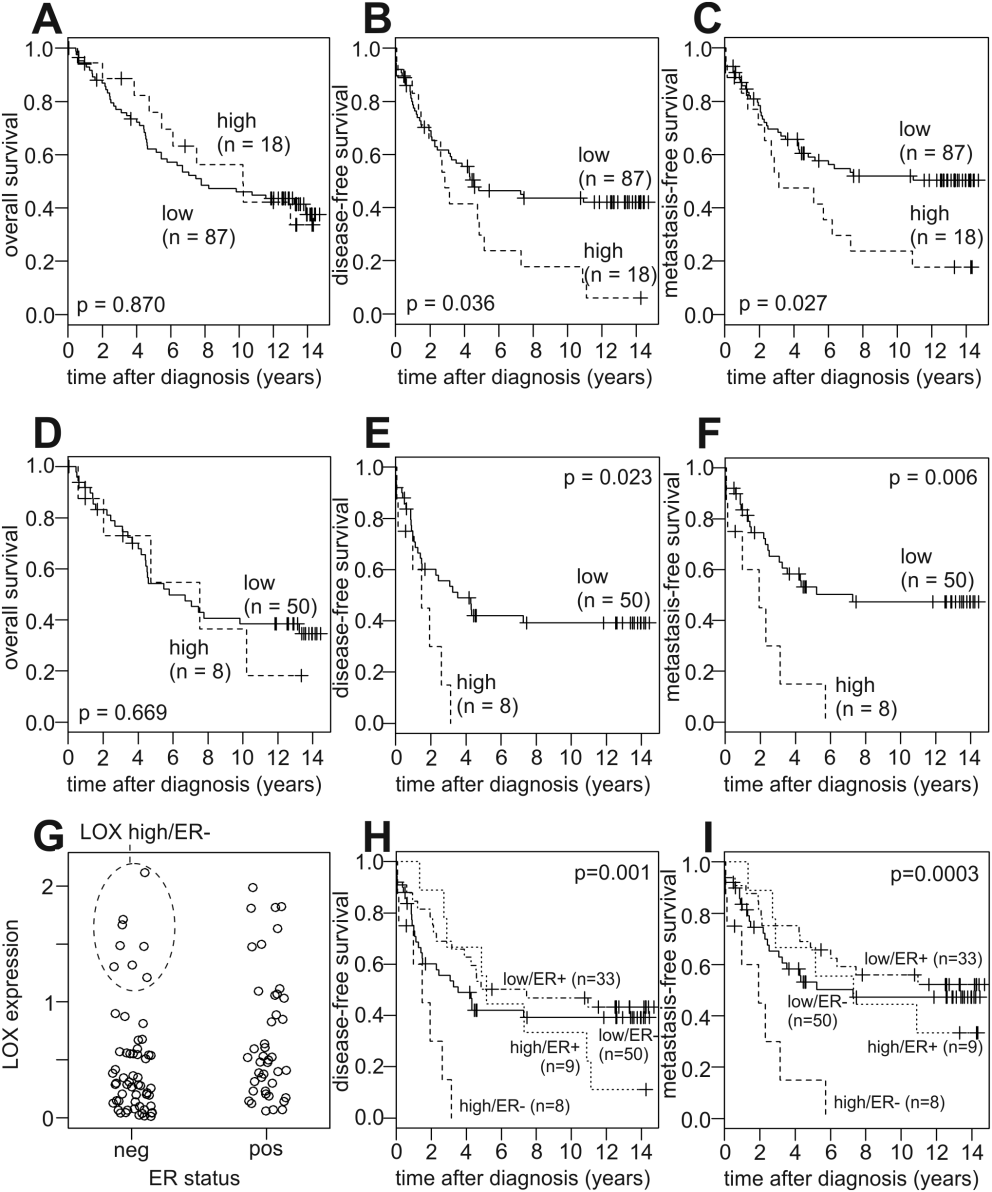
Association of LOX expression with the survival of breast cancer patients. Kaplan-Meier analyses of the overall (A, D), disease-free (B, E, H) and metastasis-free survival (C, F, I) in unse­lected patients (A–C; n = 105) and in ER-negative patients (D–F; n = 58) are shown. (G) Strip-chart of individual patients with their corresponding ER-status and relative LOX expression levels. Patients were stratified into four groups according to those two parameters: LOX high/ER−, LOX low/ER−, LOX high/ER+, and LOX low/ER+. (H, I) Kaplan-Meier analyses of the four groups established in (G). LOX high, relative LOX expression >1.094; LOX low, relative LOX expression <1.094; ER−, estrogen receptor negative; ER+, estrogen receptor positive.

Relative LOX mRNA expression was quantified in primary tumour tissue samples of 105 patients and in one lymph node metastasis each for 17 of these patients. Potential variations in LOX expression associated with well-established clinical and histopathological characteristics of breast cancer were visualized with boxplots, and their significance assessed with unpaired, two-sided t-tests (Fig. 3). LOX expression did not correlate significantly with tumour stage, tumour size, lymph node status, progesterone receptor status, HER2 status, or G473A genotype, in agreement with our analysis of breast cancer cell lines (Fig. 3AB; see above). In contrast, significantly elevated mean LOX mRNA levels were associated with an age ≥55 years (1.7±1.2-fold; p = 0.003; Fig. 3C), post-menopause (1.6±1.3-fold; p = 0.014; Fig. 3D), and p53 negativity (1.6±1.4-fold; p = 0.046; Fig. 3L). LOX mRNA levels were also elevated in estrogen receptor positive tumors (mean, 1.4±1.2-fold; median, 1.7-fold; p = 0.049; Fig. 3I). However, although the mean and median LOX expression levels in ER negative tumours were lower than in ER positive tumours, there was a distinct subgroup of ∼14% (8/58) ER negative patients whose LOX expression levels were considerably higher than in all other ER negative tumours. This subgroup stands out as outliers in the boxplot in Fig. 3I, and is also highlighted in Fig. 4G (see below). Finally, we compared LOX mRNA levels of primary tumours and lymph node metastases of 17 patients. Mean LOX mRNA levels in primary tumours were 2.7±2.5-fold higher than in corresponding, paired lymph node metastases, and the median levels were 2.3-fold higher (p = 0.002, paired t-test; Fig. 3E).

### Association of LOX Expression with Breast Cancer Prognosis

Although ER negative patients exhibited a lower mean LOX mRNA expression than ER positive patients overall, we noted that a distinct subgroup was clearly separated from the rest by its considerably higher LOX expression (highlighted in Fig. 4G; see also Fig. 3I). We defined those ∼14% (8/58) ER negative patients as LOX-high, and the other 50 patients as LOX-low. Subsequently, the same cutoff was also applied to patients with a positive or unknown ER status. 18 patients were thus considered as LOX-high (eight ER negative, nine ER positive and one whose ER status was not known), and the other 87 patients as LOX-low. We next performed Kaplan-Meier analyses of the overall (OS), disease-free (DFS), and metastasis-free survival (MFS) of LOX-high vs. LOX-low patients (Fig. 4). Unselected and ER-negative patients with a high LOX expression showed a significantly poorer disease-free- and metastasis-free survival than LOX-low patients, whereas no such effect was observed in ER-positive patients (Fig. 4; Fig. S1). Conversely, LOX expression showed no significant impact on the overall survival, neither in unselected patients nor in ER-positive or ER-negative patients (Fig. 4A, D; Fig. S1A). Thus, DFS and MFS were significantly influenced both by LOX expression and ER status. Accordingly, we stratified our study population by both these parameters into four groups, and compared their DFS and MFS. In these analyses, LOX high/ER− patients exhibited a very poor prognosis. One of these patients was lost from follow-up after 204 days, but the other seven all developed recurrent disease within 3.1 years, and metastases within 5.7 years (Fig. 4HI). Conversely, the other three groups (LOX high/ER+, LOX low/ER+ and LOX low/ER−) were comparable to each other, and had a much better prognosis than the LOX high/ER− both in the DFS (p = 0.001) and in the MFS (p = 0.0003; Fig. 4HI).

**Figure 5:**
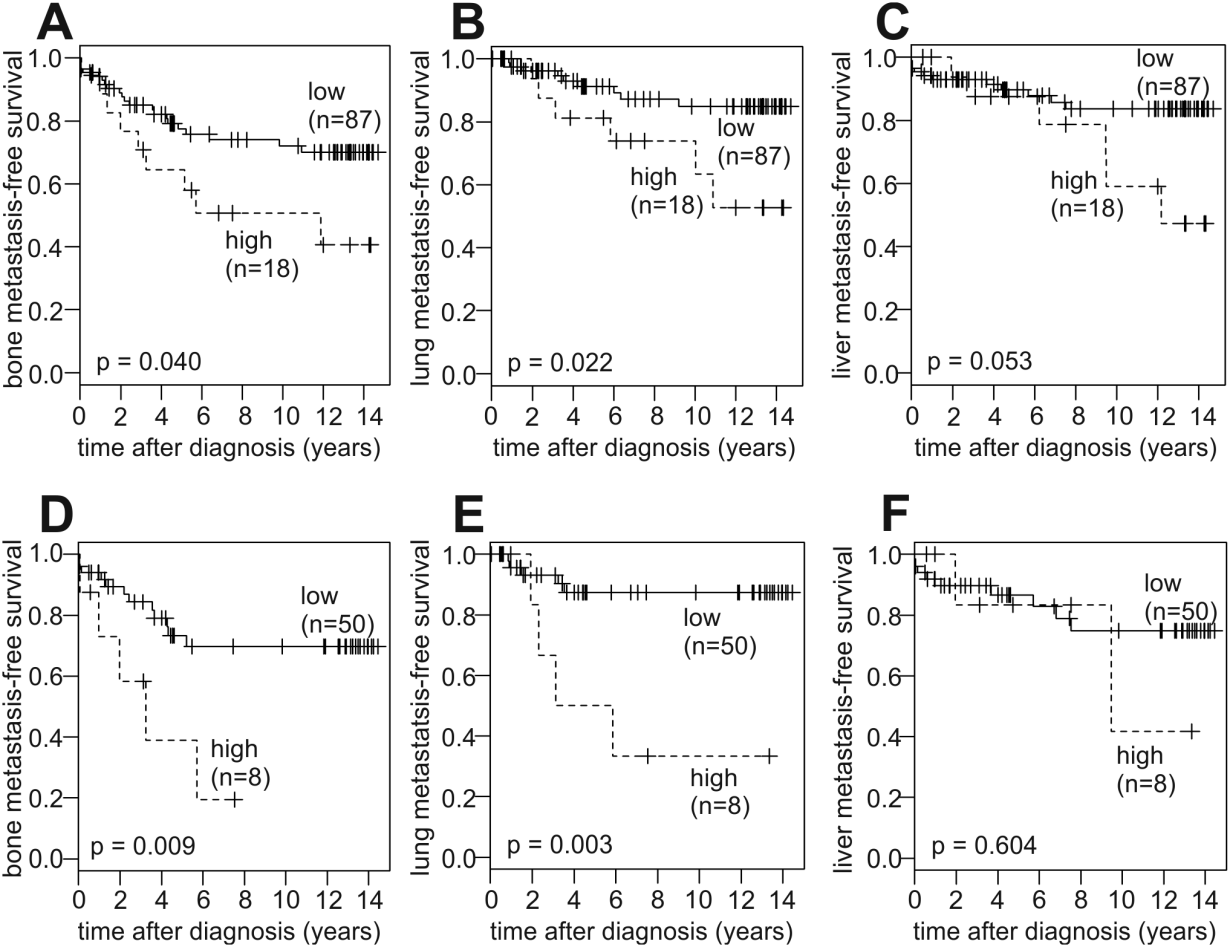
Association of LOX expression with metastasis to specific target sites. Kaplan-Meier analyses of bone- (A, D), lung- (B, E) and liver-metastasis-free survival (C, F) in unselected patients (A–C; n = 105) and in ER-negative patients (D–F; n = 58) are shown. ER, estrogen receptor.

Unselected and ER-negative patients with high LOX expression also showed a significant decrease in bone- and lung-metastasis-free survival, but not in liver-metastasis-free survival (Fig. 5A–F). In contrast, high LOX expression exhibited no significant association with bone-, lung- and liver-metastasis-free survival in ER-positive patients (Fig. S1D–F). In conclusion, patients with a high relative LOX mRNA expression exhibited a decreased survival and poor prognosis in most of these analyses. This effect was apparently limited to ER negative patients, consistent with a previous report [3]. Moreover, we found that the small subgroup of LOX high/ER negative patients (7.6% of the study population; 8/105) exhibited a very short DFS and MFS.

We next performed multivariable Cox proportional hazards regression analyses of the DFS and MFS including the variables LOX expression, G473A genotype and ER status. In a parallel univariable analysis, each variable was also analysed individually (Table S3 and S4). LOX expression was a potent independent prognostic factor in both DFS (HR, 2.21; 95% CI, 1.14–4.30; p<0.02) and MFS (HR, 2.40; 95% CI, 1.23–4.66; p<0.01). In contrast, the hazard ratios associated with the G473A genotype, which were non-significantly elevated in the parallel univariate analyses, considerably decreased in the multivariate analysis.

## Discussion

The G473A SNP affects the amino acid sequence of LOX, and the Gln variant encoded by the minor A-allele has been shown to compromise the tumour-suppressive activity of the LOX pro-peptide [4]. Obviously, such a loss-of-function substitution could lead to an increased cancer risk, and the A-allele was indeed associated with a significantly elevated breast cancer risk in a Chinese Han population [18]. In contrast, we did not find evidence for an association of the G473A SNP with breast cancer risk in the current first analysis of a European study population. Likewise, the G473A SNP was not associated with breast cancer risk in African American women [4]. Thus, the contribution of the LOX G473A SNP to breast cancer susceptibility may differ among populations [18]. Interestingly, most reported associations of the G473A SNP with risk of other cancer types were also found in Asian populations [13]–[17].

We also found that A-carriers had a significantly later mean age at breast cancer onset than patients with the GG genotype. The major difference between A-carriers and GG patients occurred at an age of 50–60, which roughly coincides with menopause. Interestingly, patients older than 55 years and post-menopausal patients also exhibited significantly elevated intra-tumoural LOX mRNA levels. In contrast, LOX mRNA levels were found progressively reduced with increasing age in the rat aorta [19]. In this study, downregulation of LOX expression was accompanied by a parallel downregulation of its substrates tropoelastin and type I collagen, whereas collagens are frequently overexpressed in carcinomas, which may explain these divergent results [1], [19], [20].

We next analysed for the first time whether the G473A SNP is associated with breast cancer prognosis. These Kaplan-Meier analyses revealed a clear trend towards a decreased disease-free survival of A-carriers in ER negative patients, although it was not significant at the p<0.05 level. A similar, but weaker trend was found for the metastasis-free survival. In contrast, no effect was observed in ER positive patients. Consistently, the AA genotype was associated with a significantly shorter survival in non-small cell lung cancer [14]. Our study had an insufficient number of AA patients for a separate analysis (n = 3), and hence we grouped them together with GA patients for survival analyses. It would be interesting to analyse the association of the AA genotype with breast cancer prognosis in future larger studies and/or meta-analyses.

High LOX expression was found to be associated with a significant decrease in metastasis-free survival in unselected patients. This association was more pronounced in ER negative patients, whereas only a weak, non-significant trend was observed in ER positive patients, consistent with a previous report [3]. We extended this analysis to the disease-free survival and obtained very similar results. We also analysed the survival free of metastasis to the bone, lung or liver, the major target organs of distant breast cancer metastases. High LOX expression was significantly associated with a poor bone- and lung-metastasis-free survival, which was again more pronounced in ER negative patients, but absent in ER positive patients. Association of high LOX expression with liver-metastasis-free survival was of borderline significance (p = 0.053). Opposite to all other survival analyses, this effect was predominantely due to ER positive cases, with only a weak trend in ER negative patients. In contrast to a previous report, LOX expression was not significantly associated with the overall survival, which may, at least in part, be due to the smaller number of patients in the present study [3]. The prognostic power of LOX expression was more pronounced with respect to metastasis-free survival than overall survival also in that previous study [3]. Finally, in multivariable Cox proportional hazards regression analyses of the DFS and MFS of the variables LOX expression, G473A genotype and ER status, LOX expression was a potent independent prognostic parameter (Table S3 and S4; see below).

High LOX expression has been associated with increased metastasis and poor prognosis in a number of different tumour types such as breast, colorectal, head and neck, and prostate cancer [3], [21], [22]. On the other hand, reduced LOX expression has also been reported in many carcinomas [4]–[7]. Here we show that LOX expression is reduced in lymph node metastases compared to matched primary tumour samples from the same patients. In contrast, no significant differences in LOX expression were found in tumours of lymph node positive vs. negative patients, arguing against a simple effect of cancer progression. We conclude that even if LOX may be highly relevant for metastasis formation, it may no longer be required in the metastasis itself once established. Moreover, the mechanism of lymph node metastasis is quite different from that of distant metastasis, in which LOX presumably has a central role [1], [23]–[25]. These LOX expression patterns may seem inconsistent, and may reflect the dual role of LOX in tumour suppression and metastatic progression.

We noted several similarities of the G473A SNP and LOX expression with respect to the association with breast cancer survival. High LOX expression was significantly associated with poor DFS and MFS in ER negative patients, whereas there was only a weak, non-significant trend in ER positive patients. Moreover, significant associations were observed with DFS and MFS but not OS. Although the A-allele was not significantly associated with prognosis, a trend of association was observed which followed an identical pattern, suggesting that these two variables are dependent. However, LOX expression was not affected by G473A genotype in tumours or breast cancer cell lines, and the genotypes of the eight ER negative/LOX high patients with the poorest prognosis (Fig. 4H; see below) were inconspicuous: five GG, three GA. Accordingly, multivariate Cox proportional hazards regression analyses of the DFS and MFS including LOX expression, G473A genotype and ER status as prognostic variables were performed. LOX expression was a potent independent prognostic parameter in these analyses. The hazard ratios associated with the G473A A-allele were non-significantly elevated in the parallel univariate analyses, but considerably decreased in the multivariate analyses, indicating that its prognostic value is not independent of LOX expression and/or ER status.

Several lines of evidence suggest a functional link of LOX with the estrogen receptor (ER) in breast cancer. First, a trend towards a dose-dependent association of the A-allele of the G473A SNP with an increased risk of ER negative, but not ER positive breast cancer was shown [4]. Likewise, we found a trend towards a poor survival of G473A A-allele carriers in ER negative, but not ER positive breast cancer patients. Moreover, the potent prognostic power of LOX expression was essentially limited to ER negative breast cancer, consistent with a previous report [3]. We and others have further shown that LOX expression is associated with the ER status in breast tumours [3], [4]. LOX expression can be induced by hypoxia via HIF1, and a hypoxia response signature identified by expression profiling is also associated with ER status in breast tumours [3], [26].

In the present study, the mean and median LOX expression levels were lower in ER negative vs. positive tumours, however, there was a clearly separated subgroup of ∼14% ER negative patients with considerably higher LOX expression levels than all other ER negatives. When we stratified our study population by both LOX expression and ER status, these LOX high/ER negative patients exhibited a very poor survival, whereas the other three subgroups (LOX high/ER+, LOX low/ER+ and LOX low/ER−) were comparable to each other, and had a much better prognosis than the LOX high/ER− patients.

In summary, we report the first analysis of the G473A SNP in a European population, in which we found no evidence for an association with an increased breast cancer risk. However, the minor A-allele was associated with an older age at breast cancer onset, and a trend towards a poorer disease-free survival, particularly in ER negative patients. We determined LOX mRNA expression by qRT-PCR in the tumours of 105 patients, which was significantly associated with the age at onset, menopausal status, ER status and p53 status. In addition, LOX expression was determined in the lymph node metastases of 17 patients, and was found to be significantly lower than in the matched primary tumours of the same patients. High LOX expression was associated with a decreased disease-free and metastasis-free survival in unselected and in ER negative patients. In particular, patients with a high LOX expression and a negative ER status constituted a distinct subgroup with a pronounced shortening of their disease-free and metastasis-free survival compared to all other patients. There are some limitations of our study, though. First, our study had insufficient cases to detect a significant differential risk in subgroup analyses. Second, since follow-up data were available for only a subset of the patients in whom the G473A SNP was genotyped, a survival bias might exist concerning the SNP analysis, particularly since the fraction of ER positive and negative patients in this subset differs from that of the entire study population. However, this does not affect the LOX expression analyses, since follow-up data were available for all those patients. Taken together, we have possibly identified a novel, biologically and clinically relevant subgroup of ER negative breast cancer patients based on LOX expression. Since ER is a routine diagnostic parameter in clinical breast cancer care, it may be worthwhile to analyse LOX expression in parallel to facilitate identification of patients belonging to this discrete subgroup with a very poor prognosis. Once identified, these patients could be eligible to more frequent and thorough follow-up examinations after their initial surgical breast cancer therapy, and/or to different treatment options. LOX itself has recently been argued to be a promising cancer therapeutic target [1], and we consider LOX high/ER negative patients as a suitable collective for the initial breast cancer trials if in place.

## Patients and Methods

### Study Population

This study was approved and is annually reviewed by the Institutional Review Board (“Ethikkommission”) of the Medical University of Vienna, Austria (MUV). 276 consecutive breast cancer patients treated between 2002 and 2004, and another 138 patients treated between 1991 and 1994 at the Department of Obstetrics and Gynecology, MUV, were enrolled in this study. From the latter 138 patients, detailed follow-up records as well as fresh-frozen tumour tissue were available, which was subjected to isolation of total RNA and/or genomic DNA. Patients with benign gynecological lesions and healthy females without any malignancies in their personal history (n = 255) were enrolled as controls between 2002 and 2004 at the Department of Obstetrics and Gynecology, MUV, and written informed consent was obtained from all participants. Only women of Western European descent from the same geographical area were included as patients or controls. Genomic DNA was isolated from 398 patients and 255 controls, and genotyping of the G473A SNP was successful for 386 patients and 243 controls. Total RNA was isolated from 111 fresh-frozen tumour samples as described [27], but for technical reasons LOX expression could only be determined in 105 of them. For 17 patients, LOX mRNA levels were also determined in one lymph node metastasis each in addition to the primary tumour. Both the G473A genotype and LOX mRNA expression was successfully determined in an overlapping set of 86 patients. Clinical and histopathological characteristics of the study population are shown in Table S1.

### DNA Isolation and Genotyping

Genomic DNA has been extracted previously from blood samples with the QIAamp DNA Blood Midi kit (Qiagen, Venlo, The Netherlands), and from 122 fresh-frozen primary tumour samples with the High Pure PCR Template Preparation Kit (Roche, Vienna, Austria) as described [27], [28]. Genotyping of SNP rs1800449 (G473A; c.473G>A; Arg158Gln; R158Q) in the *LOX* gene was performed by TaqMan PCR with Genotyping Mastermix and allele-specific, fluorescently labeled probes on a 7500 fast instrument following the manufacturer’s instructions (Applied Biosystems, Brunn/Gebirge, Austria; Assay-ID #C___7574719_10). 20 ng of genomic DNA were used per reaction in a reaction volume of 10 µl.

### qRT-PCR Analysis of LOX mRNA Expression

Isolation of total RNA with TRIreagent (Sigma), quality control with the Bioanalyser 2100 (Agilent), and reverse transcription with the high-capacity cDNA archive Kit (Applied Biosystems) have been described [27], [29]. Each sample was analysed in duplicate by quantitative reverse transcription-PCR (qRT-PCR) on an Applied Biosystems 7500 fast real-time PCR instrument, using the following gene-specific primers and fluorescent probes obtained from Applied Biosystems: LOX, hs_00952480_m1; β-actin (control), hs_99999903_m1. The mRNA levels of LOX were normalised to those of β-actin in each sample, and were further normalized to controls by setting the mean level of four samples of normal breast tissue to unity (1), and expressing the levels of all other samples relative to those. All relative LOX mRNA expression levels are presented as linear 2^−ΔΔCt^ values as described [27].

### Cell Lines

All cell lines except HMEC were purchased from American Type Culture Collection (ATCC) or “Deutsche Sammlung von Mikroorganismen und Zellkulturen” (DSMZ), and were cultivated at 37°C, 5% CO_2_, and 100% humidity as described [30]. Finite-lifespan untransformed human mammary epithelial cells (HMEC) were kindly provided by M. R. Stampfer [31] and grown in MEGM medium. Total RNA and genomic DNA were isolated from all cell lines within 10 or fewer passages after receipt. RNA isolation and quality control has been described previously [30], and genomic DNA was isolated with a High Pure PCR Template Preparation Kit (Roche, Vienna, Austria) following the manufacturer’s instructions.

### Statistical Analyses

Statistical analyses were performed with R version 2.15.1 (“Roasted Marshmallows”), an open-source language and environment for statistical computing [32]. Chi-square tests with Yates’ continuity correc­tion were used to evaluate potential deviations of the study population from Hardy-Weinberg equilib­rium. All 95% confidence intervals and p-values pertaining to odds ratios were calculated by the mid-P exact method. We consider the results of our subgroup analyses in Table 1 and Fig. 3 as explor­atory, and hence did not adjust for multiple testing, as recommended previously [33]. Differences between the indicated groups with respect to relative LOX mRNA levels as well as ages at onset were analysed by unpaired, two-sided t-tests unless indicated otherwise. Survival analyses as well as follow-up details of our study population have been described in [27]. P-values to Kaplan-Meier curves and curves of cumulative breast cancer incidence were calculated by log-rank tests as described [34]. All p-values shown are two-sided. P<0.05 was considered significant.

## Supporting Information

Figure S1 Association of *LOX* G473A genotypes and LOX expression with survival in ER positive breast cancer patients. Kaplan-Meier analyses of the overall **(A, G)**, disease-free **(B, H)** and metastasis-free **(C, I)** survival. **A–C**, as a function of LOX-expression (n = 42); **G–I**, as a function of *LOX*-genotype (n = 51) **D–F**, Kaplan-Meier analyses of the bone-, lung- and liver-metastasis-free survival as a function of LOX expression (n = 42). high, relative LOX expression >1.094; low, relative LOX expression <1.094; A-carriers, patients with the AG or AA genotype.
(TIF)

Table S1 Clinical characteristics of the study population and *LOX* G473A genotype frequencies in the indicated subpopulations.
(DOCX)

Table S2 Association of G473A genotypes and alleles with breast cancer risk.
(DOCX)

Table S3 Univariable and multivariable analyses of the disease-free survival using a Cox proportional hazards model.
(DOCX)

Table S4 Univariable and multivariable analyses of the metastasis-free survival using a Cox proportional hazards model.
(DOCX)
