# Supplementary figures and images for: Association of the G473A Polymorphism and Expression of Lysyl Oxidase with Breast Cancer Risk and Survival in European Women: A Hospital-Based Case-Control Study
Source: PLoS One. 2014 Aug 20;9(8):e105579. doi: 10.1371/journal.pone.0105579 (PMC4139364; doi:10.1371/journal.pone.0105579)

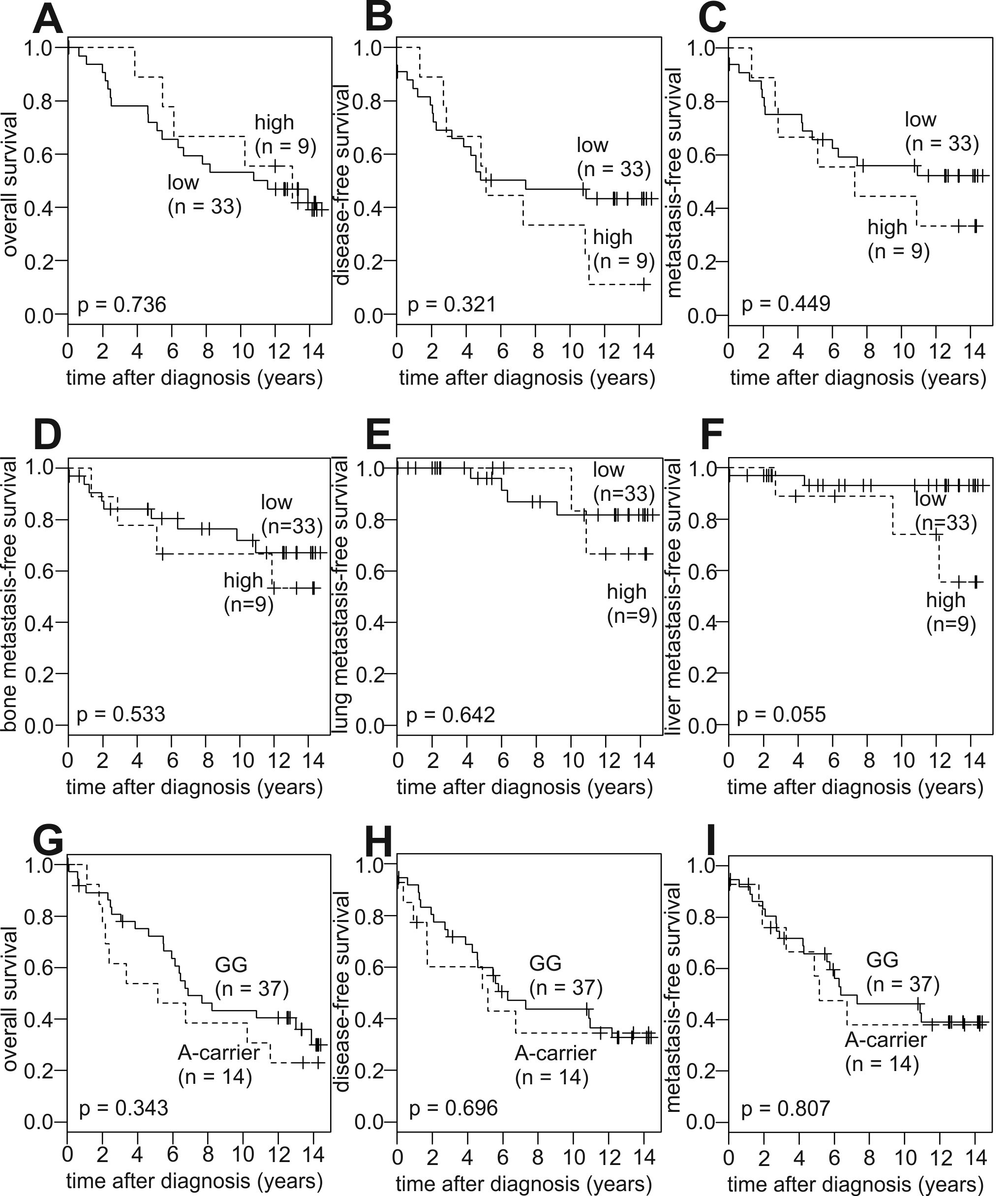

Supplement: Figure S1 — Association of LOX G473A genotypes and LOX expression with survival in ER positive breast cancer patients. Kaplan-Meier analyses of the overall (A, G), disease-free (B, H) and metastasis-free (C, I) survival. A–C, as a function of LOX-expression (n = 42); G–I, as a function of LOX-genotype (n = 51) D–F, Kaplan-Meier analyses of the bone-, lung- and liver-metastasis-free survival as a function of LOX expression (n = 42). high, relative LOX expression >1.094; low, relative LOX expression <1.094; A-carriers, patients with the AG or AA genotype. (TIF) [file pone.0105579.s001.tif]
