# Supplementary material for: Association of the G473A Polymorphism and Expression of Lysyl Oxidase with Breast Cancer Risk and Survival in European Women: A Hospital-Based Case-Control Study
Source: PLoS One. 2014 Aug 20;9(8):e105579. doi: 10.1371/journal.pone.0105579 (PMC4139364; doi:10.1371/journal.pone.0105579)
Supplement: Table S1 — Clinical characteristics of the study population and LOX G473A genotype frequencies in the indicated subpopulations. (DOCX) [file pone.0105579.s002.docx]

**Table S1.** Clinical characteristics of the study population and *LOX* G473A genotype frequencies in the indicated subpopulations.

|  |  | **Total** | **GG** | **GA** | **AA** |
| --- | --- | --- | --- | --- | --- |
| **All subjects** |  | 629 | 456 (72.5%) | 155 (24.6%) | 18 (2.9%) |
| **Patients** |  | 386 | 282 (73.1%) | 93 (24.1%) | 11 (2.8%) |
| **Controls** |  | 243 | 174 (71.6%) | 62 (25.5%) | 7 (2.9%) |
| **Patient subgroups** |  |  |  |  |  |
| **Age** | <55 | 164 | 127 (77.4%) | 33 (20.1%) | 4 (2.4%) |
|  | ≥55 | 222 | 155 (69.8%) | 60 (27.0%) | 7 (3.2%) |
| **Menopausal status** | pre | 95 | 73 (76.8%) | 21 (22.1%) | 1 (1.1%) |
|  | post | 238 | 167 (70.2%) | 63 (26.5%) | 8 (3.4%) |
|  | na | 53 | 42 (79.2%) | 21 (22.1%) | 2 (3.8%) |
| **Tumor size** | pT1 | 168 | 123 (73.2%) | 40 (23.8%) | 5 (3.0%) |
|  | pT2-4 | 149 | 104 (69.8%) | 40 (26.8%) | 5 (3.4%) |
|  | other. na | 69 | 55 (79.7%) | 13 (18.8%) | 1 (1.4%) |
| **Tumor type** | ductal | 233 | 173 (74.2%) | 54 (23.2%) | 6 (2.6%) |
|  | lobular | 78 | 51 (65.4%) | 25 (32.1%) | 2 (2.6%) |
|  | other. na | 75 | 58 (77.3%) | 14 (18.7%) | 3 (4.0%) |
| **Stage** | 0 or I | 135 | 104 (77.0%) | 28 (20.7%) | 3 (2.2%) |
|  | II-IV | 178 | 125 (70.2%) | 47 (26.4%) | 6 (3.4%) |
|  | other. na | 73 | 53 (72.6%) | 18 (24.7%) | 2 (2.7%) |
| **Grade** | pG1-2 | 226 | 167 (73.9%) | 53 (23.5%) | 6 (2.7%) |
|  | pG3 | 138 | 102 (73.9%) | 31 (22.5%) | 5 (3.6%) |
|  | na | 22 | 13 (59.1%) | 9 (40.9%) | 0 (0.0%) |
| **Lymph node status** | pN0 | 184 | 138 (75.0%) | 42 (22.8%) | 4 (2.2%) |
|  | pN+ | 126 | 91 (72.2%) | 31 (24.6%) | 4 (3.2%) |
|  | na | 76 | 53 (69.7%) | 20 (26.3%) | 3 (3.9%) |
| **ER status** | pos | 232 | 165 (71.1%) | 61 (26.3%) | 6 (2.6%) |
|  | neg | 111 | 83 (74.8%) | 24 (21.6%) | 4 (3.6%) |
|  | na | 43 | 34 (79.1%) | 8 (18.6%) | 1 (2.3%) |
| **PR status** | pos | 160 | 115 (71.9%) | 40 (25.0%) | 5 (3.1%) |
|  | neg | 177 | 129 (72.9%) | 43 (24.3%) | 5 (2.8%) |
|  | na | 49 | 38 (77.6%) | 10 (20.4%) | 1 (2.0%) |
| **HER2 status** | pos | 68 | 46 (67.6%) | 20 (29.4%) | 2 (2.9%) |
|  | neg | 272 | 200 (73.5%) | 64 (23.5%) | 8 (2.9%) |
|  | na | 46 | 36 (78.3%) | 9 (19.6%) | 1 (2.2%) |
| **p53 status** | pos | 88 | 70 (79.5%) | 16 (18.2%) | 2 (2.3%) |
|  | neg | 249 | 174 (69.9%) | 67 (26.9%) | 8 (3.2%) |
|  | na | 49 | 38 (77.6%) | 10 (20.4%) | 1 (2.0%) |
| **KI67 pos cells** | ≤10% | 175 | 123 (70.3%) | 48 (27.4%) | 4 (2.3%) |
|  | >10% | 120 | 87 (72.5%) | 29 (24.2%) | 4 (3.3%) |
|  | na | 90 | 71 (78.9%) | 16 (17.8%) | 3 (3.3%) |

Numbers of patients in the indicated subgroups are shown. Parenthesized numbers show the fraction of patients (%) with the indicated genotypes. ER, estrogen receptor; PR, progesterone receptor; na, status not available.
