# Supplementary material for: Association of the G473A Polymorphism and Expression of Lysyl Oxidase with Breast Cancer Risk and Survival in European Women: A Hospital-Based Case-Control Study
Source: PLoS One. 2014 Aug 20;9(8):e105579. doi: 10.1371/journal.pone.0105579 (PMC4139364; doi:10.1371/journal.pone.0105579)
Supplement: Table S2 — Association of G473A genotypes and alleles with breast cancer risk. (DOCX) [file pone.0105579.s003.docx]

**Table S2.** Association of G473A genotypes and alleles with breast cancer risk.

|  | **unadjusted** | | | **Adjusted^a^** | | |
| --- | --- | --- | --- | --- | --- | --- |
| **Genotypes/Alleles** | **OR** | **95% CI** | **p-value** | **OR** | **95% CI** | **p-value** |
| **AA vs. GG** | 0.97 | 0.37 - 2.55 | 0.950 | 1.02 | 0.32 - 3.28 | 0.992 |
| **AA vs. GA** | 1.05 | 0.39 - 2.85 | 0.927 | 1.41 | 0.43 - 4.67 | 0.572 |
| **GA vs. GG** | 0.93 | 0.64 - 1.34 | 0.684 | 0.73 | 0.47 - 1.13 | 0,159 |
| **AA + GA vs. GG** | 0.93 | 0.65 - 1.33 | 0.692 | 0.76 | 0.50 - 1.15 | 0.191 |
| **AA vs. GA + GG** | 0.99 | 0.38 - 2.59 | 0.982 | 1.10 | 0.35 - 3.52 | 0.866 |
| **A vs. G** | 0.95 | 0.70 - 1.29 | 0.727 | 0.82 | 0.57 - 1.18 | 0.281 |

Results of analyses of breast cancer cases vs. controls of the indicated genotypes are shown. ^a^adjusted for age and menopausal status. OR, odds ratios; 95% CI, 95% confidence intervals.
