# Supplementary material for: Association of the G473A Polymorphism and Expression of Lysyl Oxidase with Breast Cancer Risk and Survival in European Women: A Hospital-Based Case-Control Study
Source: PLoS One. 2014 Aug 20;9(8):e105579. doi: 10.1371/journal.pone.0105579 (PMC4139364; doi:10.1371/journal.pone.0105579)
Supplement: Table S3 — Univariable and multivariable analyses of the disease-free survival using a Cox proportional hazards model. (DOCX) [file pone.0105579.s004.docx]

**Table S3.** Univariable and multivariable analyses of the disease-free survival using a Cox proportional hazards model.

|  | | **univariable** | | | **multivariable** | | |
| --- | --- | --- | --- | --- | --- | --- | --- |
| **Variable** | **Subcategory** | **HR** | **95% CI** | **p-value** | **HR** | **95% CI** | **p-value** |
| **G473A genotype** | GG = 0, A‑carrier = 1 | 1.50 | 0.87-2.59 | 0.1440 | 1.19 | 0.63-2.24 | 0.6000 |
| **LOX expression** | low = 0, high = 1 | 1.82 | 1.03-3.21 | 0.0403 | 2.21 | 1.14-4.30 | 0.0190 |
| **ER status** | pos = 0, neg = 1 | 1.23 | 0.79-1.92 | 0.3700 | 1.79 | 0.99-3.26 | 0.0561 |

HR, hazard ratio; 95% CI, 95% confidence intervals; ER, estrogen receptor.
