# Supplementary material for: Association of the G473A Polymorphism and Expression of Lysyl Oxidase with Breast Cancer Risk and Survival in European Women: A Hospital-Based Case-Control Study
Source: PLoS One. 2014 Aug 20;9(8):e105579. doi: 10.1371/journal.pone.0105579 (PMC4139364; doi:10.1371/journal.pone.0105579)
Supplement: Table S4 — Univariable and multivariable analyses of the metastasis-free survival using a Cox proportional hazards model. (DOCX) [file pone.0105579.s005.docx]

**Table S4.** Univariable and multivariable analyses of the metastasis-free survival using a Cox proportional hazards model.

|  | | **univariable** | | | **multivariable** | | | |
| --- | --- | --- | --- | --- | --- | --- | --- | --- |
| **Variable** | **Subcategory** | **HR** | **95% CI** | **p-value** | **HR** | **95% CI** | **p-value** |  |
| **G473A genotype** | GG = 0, A‑carrier = 1 | 1.36 | 0.75-2.45 | 0.3140 | 1.00 | 0.51-1.97 | 0.9992 |  |
| **LOX expression** | low = 0, high = 1 | 1.97 | 1.07-3.63 | 0.0304 | 2.40 | 1.23-4.66 | 0.0099 |  |
| **ER status** | pos = 0, neg = 1 | 1.26 | 0.77-2.07 | 0.3610 | 1.47 | 0.80-2.69 | 0.2157 |  |

HR, hazard ratio; 95% CI, 95% confidence intervals; ER, estrogen receptor.
